# Spanish Adaptation of the Inventory Brief Child Abuse Potential and the Protective Factors Survey

**DOI:** 10.3389/fpsyg.2021.705228

**Published:** 2021-08-03

**Authors:** Arturo Sahagún-Morales, Amada Ampudia Rueda, Salvador Chacón-Moscoso, Susana Sanduvete-Chaves, Ennio Héctor Carro Pérez, Patricia Andrade Palos

**Affiliations:** ^1^Facultad de Psicología, Universidad Nacional Autónoma de Mexico, Ciudad de Mexico, Mexico; ^2^Departamento de Psicología Experimental, Universidad de Sevilla, Sevilla, Spain; ^3^Departamento de Psicología, Universidad Autónoma de Chile, Santiago de Chile, Chile; ^4^Facultad de Derecho y Ciencias Sociales, Centro de Investigación y Desarrollo Tecnológico Aplicado al Comportamiento, Universidad Autónoma de Tamaulipas, Tampico, Mexico

**Keywords:** validity evidences, reliability, norms and interpretation of tests scores, child abuse, protective and risk factors

## Abstract

Child maltreatment is a public health problem with different consequences depending on the form of abuse. Measuring risk and protective factors has been a fertile ground for research, without involving instruments with sufficient evidence of validity. The aim of the study was to gather evidence of validity and reliability of the Inventory Brief Child Abuse Potential (IBCAP) and Protective Factors Survey (PFS) in the Mexican population. The instruments were translated into Spanish. In a non-probabilistic sample of 200 participants, the 7-factor model for the IBCAP [comparative fit index (CFI) = 0.984; root mean square error of approximation (RMSEA) = 0.067] and the 4-factor model for the PFS (CFI = 0.974; RMSEA = 0.061) were confirmed, showing adequate fit indices. Reliability was estimated and evidence of convergent, divergent, and discriminant validity was collected, controlling for effects of social desirability. We also report interpretability statistics of the scores. We achieved solid progress in the development of instrumentation that allows determining the presence or absence of protective and risk factors for child abuse.

## Introduction

The World Health Organization defines child abuse as all forms of physical and/or emotional ill-treatment, sexual abuse, neglect, or negligent treatment or commercial or other exploitation, resulting in actual or potential harm to the health, survival, development or dignity of a child in the context of a relationship of responsibility, trust, or power [Organización Panamericana de la Salud (OPS) Oficina Regional para las Américas de la Organización Mundial de la Salud (OMS), 2003, p. 65], being the most widely used definition worldwide (Chahine, 2014; Weibela et al., 2017; Assink et al., 2018; Hayes and O'Neal, 2018; Cicchetti and Handley, 2019; Kaufman and Torbey, 2019; Marco et al., 2019; Sigad et al., 2019).

Studies point to physical abuse as a form of child abuse, which is prevalent in the world (Kessler et al., 2010). However, estimates vary according to the measurement methodologies used. Regarding its prevalence, self-reported physical abuse records 226 victims per 1,000 boys and girls, with no differences in prevalence by sex (Stoltenborgh et al., 2013). Sexual abuse is the most studied form of child abuse and its prevalence by sex worldwide records 180 victims per 1,000 girls and 76 per 1,000 boys (Stoltenborgh et al., 2011, p. 89). There is little information on the prevalence of emotional abuse compared to physical and sexual abuse [Organización Panamericana de la Salud (OPS) Oficina Regional para las Américas de la Organización Mundial de la Salud (OMS), 2003]; however, the self-reported prevalence of emotional abuse is found to be 363 victims per 1,000 boys and girls (Stoltenborgh et al., 2012a). In prevalence by sex, 363 victims of emotional abuse are reported for every 1,000 boys and 384 for every 1,000 girls (Stoltenborgh et al., 2012a). On the other hand, Stoltenborgh et al. (2012b) reported that only 16 scientific studies have recorded the self-reported prevalence. The worldwide prevalence of child abuse is found to be 163 self-reported victims per 1,000 children in physical neglect, and 184 victims per 1,000 children in emotional neglect (Stoltenborgh et al., 2012b).

In Mexico, the System for the Integral Development of the Family conducted in 2014, at the national and state level, an average of 152 children and adolescents for probable cases of child abuse, of which 35% correspond to abuse physical, 27% to neglect of care, 18% to emotional abuse, 15% to abandonment, and 4% to sexual abuse (COMPREVNNA, 2017). The same year, the National Institute of Statistics and Geography (INEGI) reported that 83% of the victims of child violence between the ages of 12 and 17 had as a perpetrator a person known as members of the household, partner, classmates and work, family, close friends, or acquaintances by sight (INEGI, 2016). Between 2010 and 2014, the main victims of child homicide were men aged from 15 to 17 years (INEGI, 2016).

The consequences of child abuse vary according to the form of abuse; in addition, there are consequences due to multiple forms of abuse. The OMS (2016) reports that child abuse is a cause of stress and is associated with early brain development disorders. In adults who have been abused in childhood, there is a greater risk of suffering and committing acts of violence, suffering depression and obesity, consuming snuff, showing sexual high-risk behavior, unwanted pregnancies, alcohol and excessive drugs, among others behavioral, physical, and mental problems. Therefore, child abuse indirectly contributes to heart disease, cancer, suicide, and sexually transmitted infections (OMS, 2016).

In general, abuse is a risk factor for a wide range of psychiatric disorders, substance abuse, behavioral problems, physical and emotional health problems, decreased well-being, propensity to commit child abuse, impaired cognitive and emotional development in children, feelings of hopelessness, low self-esteem, low self-esteem, low satisfaction with life, low sense of social support, and attachment style problems (Kessler et al., 2010; Stoltenborgh et al., 2011, 2012a, 2013, 2014; INEGI, 2016; Weibela et al., 2017; Kaufman and Torbey, 2019; Liel et al., 2019).

Taking into account the different existing definitions of child abuse that hinder the collection of verifiable information, it is considered that the official figures understate (between 50 and 80% of cases of child maltreatment are not recorded) the real prevalence of abuse (Schwab-Reese et al., 2018), so it is important to study the associated factors, both in terms of increased risk and protective factors.

Protective factors of child abuse are defined as “characteristics of a family or relationship that reduces the likelihood of child maltreatment” (Sprague-Jones et al., 2019, p. 122). In contrast, the potential factors for child abuse, or risk factors, are understood as the characteristics of a person, environment, or society that increase the probability of occurrence of child abuse (Aschengrau and Seage, 2019). Both protective and risk factors for child abuse include a wide range of environmental characteristics (physical and social), behaviors, thoughts, beliefs, and attitudes occurring in the context of a relationship, which regulate the behaviors of the members of this relationship, in such a way that they are more or less likely to commit, voluntarily or involuntarily, acts that mistreat a minor.

Studies have identified recurrent risk and protective factors for child maltreatment (McCoy and Keen, 2014). Family functioning (Thornock et al., 2019), parental relationship (McCoy and Keen, 2014), preparation of parents in parenting strategies and parental knowledge (Albertos et al., 2016; Morrongiello et al., 2019), parental values (McCoy and Keen, 2014), the participation of the child in family activities (McCoy and Keen, 2014), social support (Cutrona et al., 1994; Piko, 2000), and even community environments and characteristics of the physical properties of the home (Labella and Masten, 2018) are some of the most important protective factors (McCoy and Keen, 2014). In terms of risk factors, poverty (Delgado, 2016), family stress (Musitu and Callejas, 2017), family and intimate partner violence (Henry, 2018; Lawson, 2019), among others have been reported (McCoy and Keen, 2014).

Measuring risk and protective factors have been fertile grounds for research, without implying these instruments with sufficient validity evidence. In this case, we worked with the second edition of the Protective Factors Survey (PFS; Sprague-Jones et al., 2019) and the Inventory Brief Child Abuse Potential (IBCAP; Ellonen et al., 2019).

The IBCAP is a self-report instrument developed by Ondersma et al. (2005) from the Inventory Child Abuse Potential (ICAI; Milner, 1986). It is answered using dichotomous items of agreement/disagreement. It is a brief inventory that includes 24 items for the risk factor, scales, plus nine items for the ICAI validity scales. Stability has been reported in the factors that make up the IBCAP, showing, in the US population (Ondersma et al., 2005), a structure of seven factors, which include: Distress, Family Conflict, Rigidity, Happiness, Feelings or persecution, Loneliness and Financial insecurity. Likewise, the version by Ondersma et al. (2005) maintains the scale of lies and random response (validity scales) of the ICAI. Although the IBCAP shows acceptable validity evidences in its different versions (Ondersma et al., 2005; Ellonen et al., 2019; Liel et al., 2019), more validity evidences are required that we will seek to collect in this study.

For its part, the PFS was developed in 2005 by the FRIENDS National Center in collaboration with the Institute for Educational Research and Public Service at the University of Kansas (FRIENDS National Center for Community Based Child Abuse Prevention, 2021). The creation of PFS responded to the need for a reliable and valid instrument for the evaluation of child abuse prevention programs, given that at that time, there was no adequate instrument for measuring changes in multiple protective factors for child abuse and neglect (Sprague-Jones et al., 2019). The PFS has 20 items in 7-point Likert scale and is designed for caregivers of minors, users of prevention of child abuse services. It has a traditional version (non-retrospective self-report) and a retrospective response version and measures the factors: (a) Family Functioning and Resilience, (b) Social Supports, (c) Concrete Supports, and (d) Nurturing and Attachment; in addition to items that indicate knowledge of the development of parenting and child together without enough features to speak of a latent factor. All factors have a good reliability (FRIENDS National Center for Community Based Child Abuse Prevention, 2020).

Starting with the first PFS, a Spanish short version has been developed (for the Latino population residing in the United States, Conrad-Hiebner et al., 2015), and the second edition was also in retrospective and non-retrospective self-report format (Sprague-Jones et al., 2019). Likewise, the relationship of PFS with instruments like the Perceived Stress Scale (PSS), the PRIME-MD Patient Health Questionnaire and the same IBCAP (Counts et al., 2010) has been tested. The second edition of the PFS has 29 items in 5-point Likert scale and measures the following factors: (a) Family Functioning and Resilience, (b) Social Supports, (c) Concrete Supports, (d) Nurturing and Attachment and (e) Caregiver/Practitioner Relationship, this last factor being the only one with poor internal consistency (FRIENDS National Center for Community Based Child Abuse Prevention, 2018); although there is a more recent version and with better levels of internal consistency (Sprague-Jones et al., 2019), this remains precisely as the one used in this study.

In both the IBCAP and the PFS, the psychometric analyzes are limited to the internal consistency determined with the Cronbach's Alpha coefficient and the Exploratory Factor Analysis (EFA). This aspect is remarkable because they are insufficient and inadequate to determine the reliability and validity of an instrument (Batista-Foguet et al., 2004; Agbo, 2010). Cronbach's Alpha coefficient adequately estimates only the true internal consistency when the items are at least tau-equivalents, assuming that it is not tested and that it is practically impossible to fulfill, in addition to the fact that unidimensionality is required, which is not fulfilled in multidimensional scales (Contreras-Espinoza and Novoa-Muñoz, 2018). In the EFA, the euphemism for rotation (Batista-Foguet et al., 2004) is an arbitrary element in the decision about matching the items to the latent factor, leading to different interpretations of the same analysis according to the rotation method factor chosen. Another methodological flaw lies in assuming continuity in items that are inherently ordinal (Hoffmann et al., 2013), leading to an indiscriminate use of statistical methods involving measurement levels above the ordinal as Pearson's correlation.

Either the validation studies do not present evidence or they only present correlation matrices between variables of a nomological network without controlling for social desirability effects (Mikulic et al., 2016) or reliability attenuation effects (Domínguez-Lara, 2017) while that with regard to discrimination by item and discriminant validity, there are no indicators that demonstrate them. Finally, although both instruments have versions in different languages, there is no version that presents validity or reliability indices in the Mexican population, a crucial aspect considering that its use is common in child abuse prevention programs (Chacón-Moscoso et al., 2016, 2019).

Therefore, this paper aims to gather evidence of validity and reliability of the IBCAP and PFS in the Mexican population, resolving faults present in the previous psychometric studies.

## Materials and Methods

### IBCAP and PFS Spanish Translation Study

#### Participants

A non-probabilistic intentional sample was used. We worked with three translators whose native language is Spanish. The first translator is an expert translator, the second is a licensed psychologist with experience in working with children and parents, and the third is a Doctor of Psychology with experience in measuring the psychological evaluation. Everyone worked independently, without knowing the research objectives to maintain masked the process. Additionally, there was an evaluator of the translations who has experience in the development of psychological measurement instruments.

#### Instruments

##### Inventory Brief Child Abuse Potential

The IBCAP (Ellonen et al., 2019) consists of 21 items divided into five factors: *Loneliness and distress* (LD, nine items), *Impact of others* (IO, four items), *Family conflict* (FC, three items), *Rigidity* (R, three items), and *Financial insecurity* (FI, two items). Here the Finnish version which responds by dichotomous items of agreement/disagreement and which has a total Cronbach's Alpha of 0.781 was used for its adaptation.

##### Protective Factors Survey

The PFS (Sprague-Jones et al., 2019) consists of 29 items distributed into five factors: *Family Functioning and Resilience* (FFR, four items), *Nurturing and Attachment* (NA, seven items), *Social Supports* (SS, seven items), *Concrete Supports* (CS, eight items), and *Caregiver/Practitioner Relationship* (CPR, three items). It is a self-report instrument that is answered through 5-point Likert-type items with labels of 1 = not at all like my life, 2 = not much like my life, 3 = somewhat like my life, 4 = quite a lot like my life, and 5 = just like my life, for the FFR, NA, and SS factors respectively; of 1 = never, 2 = rarely, 3 = sometimes, 4 = often, and 5 = almost always for the CS factor; and 1 = strongly agree, 2 = agree, 3 = neither agree nor disagree, 4 = disagree, and 5 = strongly disagree for the CPR factor. Here, the American version of Sprague-Jones et al. (2019) which explains 54.1% of variance and has Cronbach's aalpha >0.750, was used for its adaptation. It was decided not to use the Spanish short version by Conrad-Hiebner et al. (2015) because, despite having been validated in the Spanish-speaking population, it only has 15 items, an aspect that limits the use of the tool in the evaluation at the individual level due to the high impact of the standard error of measurement (SEM) on short instruments (Sijtsma, 2011). Added to the above is the fact that the validation study was developed in the residents of the United States, a fact that implies important cultural differences within the population living in Mexico.

##### Format for Translation

Translation format was developed with 21 items of the IBCAP (Ellonen et al., 2019) and 29 of the PFS (Sprague-Jones et al., 2019). This instrument is the one that was presented to the translators for the translation of all items. It consists of three columns, one where the original English version, one for the translators to place their version translated into Spanish and another column is placed where the translators make observations about each item if they deem it necessary.

#### Procedure

##### Translation Process

Although the use of backward translation design is common, it has been documented that this design frequently generates translations in the target language (Spanish, in this case) that facilitate a reverse translation but do not maximize the suitability of the translation to the target population (International Test Commission, 2017). Considering this disadvantage, a forward translation design with multiple translators and subsequent revision was chosen (Muñiz et al., 2013; Hambleton and Patsula, 2014) because it allows for identifying and eliminating discrepancies between the different direct translations and creating a single version in the target language (International Test Commission, 2017). Translators were contacted *via* e-mail and the translation form was sent. Translations were performed over a period of 17–33 calendar days. The translators were asked to translate each item from English into Spanish, prioritizing meaning over literality. It was specified to all that the Spanish version should have the colloquial language.

##### Translation Evaluation and Selection Process

Concluded translations were compared with the original English version to evaluate and select the best translations. This task was performed by a psychologist with expertise in the subject of child abuse (author of this work) without prior knowledge of the identity of the persons who carried out the translation. He ruled out, one by one, each translation of the 50 items (150 translations in total) choosing the one he considered the best. The reviewers could choose one of the following options: Translation 1 is better, Translation 2 is better, Translation 3 is better, Translations 1 and 2 are better, Translations 1 and 3 are better, Translations 2 and 3 are better, All three translations are just as good.

##### Item Writing Process From Translations

With selected translations, drafts of the items of the IBCAP and PFS were developed. The writing consisted of using the terms of the selected translations to write a version that kept the meaning of the original item. At this stage, adaptations of the items to be applicable to people were performed with and without children, and to be answered using the same scale of responses (e.g., 7-point Likert scale). Also, sometimes several items were drawn from a single item because the original version contained more than an idea, something that could generate confusion among respondents.

#### Study Results of Spanish Translation

In the translation process, the IBCAP proceeded from 21 to 30 items. After translating the Finnish version of Ellonen et al. (2019), one of the translators recommended using the German version of Liel et al. (2019) as well. It was decided to comply with the recommendation because both the versions have the most recent validation studies up to the moment of doing this research, in addition to sharing 76.19% (16) of the items (the five items that were exclusively part of the German version were translated by the first author of this study focusing on the functional rather than on the literal equivalence and avoiding cultural references, idiosyncratic items, and inadequate response formats as recommended by the International Test Commission, 2017). Therefore, to the 21 items of the Finnish version of Ellonen et al. (2019), translated by the panel of translators (Muñiz et al., 2013; Hambleton and Patsula, 2014), the 5 items of the German version of Liel et al. (2019), translated by the first author of this paper, were added. The integration of both the versions resulted in a 7-factor theoretical structure in which the Impact of Others, Family Conflict, and Rigidity factors of the Finnish version remained intact, but the Loneliness and Distress factor (LD, nine items) was separated into Loneliness (L, four items) and Distress (D, four items) factors, in addition to the Unhappiness factor (U, three items) which was only found in the German version of Liel et al. (2019). Furthermore, when integrating both versions, the Financial Insecurity (FI) factor was made up of a single item, which is why three items were created directly in Spanish that complemented the factor; these items were developed by the first author of this paper. The resulting seven factors are consistent with the original version of Milner (1986). Translations and changes of the two original English versions of the IBCAP and preliminary Spanish version are detailed in Appendix A.

In the case of PFS, it proceeded from 29 to 49 items, but the original 5-factor structure of Sprague-Jones et al. (2019) was maintained. It is also possible to find all the translation details and modifications made in Appendix A.

### Study of Evidence of Validity and Reliability of the IBCAP and PFS

#### Participants

An accidental non-probabilistic sample was used (Kerlinger and Lee, 2002). The sample size was determined in 200 participants because it is an amount necessary to obtain classic statistical items as well as a stable correlation matrix for the development of factor analysis (Downing and Haladyna, 2006). Because it was sought to work with a general population, the only inclusion criteria were that the participants were between 18 and 65 years and were residing in Mexico at the time of research. There were no misses in the sample during the development of the research. The sociodemographic characteristics of the sample are presented in Table 1 and the structural characteristics of the families are presented in Appendix B.

**Table 1:** Sociodemographic characteristics of the participants (*N* = 200).

**Characteristic**	**f/M**	**%/SD**	**Characteristic**	**f**	**%**	**Characteristic**	**f**	**%**
People in the same home	3.84	1.83	Maximum degree of study			Total Monthly Income		
Age	31.79	13.12	Incomplete or in-process high school	3	1.5	Between $0 and 2,699	17	8.5
Sex			Complete high school	22	11	Between $2,700 and 6,799	47	23.5
Men	44	22	Incomplete or in-process bachelor's degree	75	37.5	Between $6,800 and 11,599	60	30
Women	156	78	Completed bachelor's degree	59	29.5	Between $11,600 and 34,999	64	32
Children			Incomplete or in-process specialty	2	1	Between $35,000 and 84,999	11	5.5
Yes	68	34	Completed specialty	4	2	$85,000 or more	1	0.5
Do not	132	66	Incomplete or in-process mastery	11	5.5			
Marital status			Complete mastery	14	7			
Married	39	19.5	Incomplete or in the process PhD	7	3.5			
Divorced	7	3.5	Complete PhD	3	1.5			
Single	129	64.5	History of alcohol / drug abuse					
Free Union	22	11	Do not	186	93			
Widower	3	1.5	Yes	14	7			

*f, absolute frequency; %, relative frequency; M, mean; SD, standard deviation*.

#### Instruments

##### Inventory Brief Child Abuse Potential Translated

The translated version of the IBCAP developed in the previous phase was used. It is made up of 30 items distributed in seven factors: *Loneliness* (L, six items), *Distress* (D, four items), *Impact of Others* (IO, four items), *Family Conflict* (FC, four items), *Rigidity* (R, four items), *Financial Insecurity* (FI, five items), and *Unhappiness* (U, three items). The response options were adjusted to seven points from 1 (*Total disagreement*) to 7 (*Total agreement*).

##### Protective Factors Survey Translated

The translated version of the PFS developed in the previous phase was used. It is made up of 49 items divided into five factors, which include: FFR, four items; NA, seven items; SS, 15 items; CS, 20 items; CPR, three items. The response options for the different factors were standardized on a 7-point scale from 1 (*Total disagreement*) to 7 (*Total agreement*), although in 13 items of the CS factor, the option, not applicable was also added.

##### Balanced Inventory of Desirable Responding

To control the effects of social desirability, the BIDR (Mikulic et al., 2016) was used. The BIDR consists of 18 items that make up a single factor, *Social Desirability* (SDes). It is a self-report instrument that is answered by Likert-type items with seven points from 1 (*False*) to 7 (*True*). In this study, the Spanish version of Mikulic et al. (2016) was validated using Confirmatory Factor Analysis (CFA) with polychoric correlations (Holgado-Tello et al., 2008; Brown, 2015; Desjardins and Bulut, 2018) and estimation of unweighted least squares with robust standard errors and test statistic adjusted to the mean (ULSM; Shi et al., 2018). The results of the validation of the BIDR are presented in this section because they are not part of the central objective of the research, but correspond to a secondary analysis, that is necessary for the fulfillment of the objectives. It was obtained a reduced version (nine items) with good fit [χ^2^ (26) = 38.605, *p* = 0.053; χ2/df = 1.485; CFI = 0.987; TLI = 0.982; RMSEA = 0.049, 95% CI (0.000, 0.090), *p* = 0.466; SRMR = 0.049] in a two-factor model (*Self-deception* and *Printing Handling* factors), such as that found in the Mexican population by Moral de la Rubia et al. (2012). In this study, evidence of convergent validity was obtained through the average variance extracted (AVE) of the Factors ≥ 0.500 (Fornell and Larcker, 1981; Cheung and Wang, 2017) as well as the factor loadings (λ) ≥ 0.500 (Cheung and Wang, 2017); evidence of discriminant validity using the r_between−factors_ ≤ 0.700 (Cheung and Wang, 2017) and the ${\text{r}}_{\text{betweenfactors}}^{2}$ < AVE (Fornell and Larcker, 1981); evidence of discrimination by item with the corrected total-element correlation, (*r*_tec_) > 0.200 (Abad et al., 2011); and evidence of total internal consistency and by factors with the coefficients, α_Ordinal_, ω_Ordinal_, and GLB_Ordinal_ > 0.700 (Trizano-Hermosilla and Alvarado, 2016; George and Mallery, 2017, see full psychometric properties of Spanish version of BIDR-9 in Appendix C).

#### Procedure

For reasons of the quarantine due to the Covid-19 pandemic, the instruments were applied *via* Google Forms. Digital forms were distributed in 19 states of Mexico using Facebook Ads service (https://www.facebook.com/permalink.php?story_fbid=104765114762260&id=104716831433755). This system allows sampling by establishing diffusion points in the states of the Mexican Republic with high population density or that are physically very distant from each other, such as Nuevo León and Yucatán, for example. Responses were collected over a period of 31 calendar days. The form included an informed consent and confidentiality statement. The study design was non-experimental, single-group, and cross-sectional.

#### Data Analysis

##### Validity Evidence Concerning the Internal Structure of the Instrument

Confirmatory factor analysis taking the matrix, polychoric correlations (Holgado-Tello et al., 2008 Brown, 2015; Desjardins and Bulut, 2018) was used. The estimation method used unweighted least squares with robust standard errors and test statistic adjusted to the mean (ULSM, Shi et al., 2018) due to the lack of multivariate normality (negative Mardia test, Porras, 2016). For the IBCAP-T a structure of seven correlated latent variables was tested, while in the PFS-T a structure of five correlated latent variables was tested. Correlated factor structures were tested in both the IBCAP-T and the PFS-T because the theoretical background suggests that the structures of both constructs are not independent (Ellonen et al., 2019; Liel et al., 2019; Sprague-Jones et al., 2019). Structures with the independent factors were also tested as rival models. The fit was evaluated using the following fit indices and interpretation criteria (Abad et al., 2011; Kline, 2011): Chi square/degrees of freedom (χ^2^/df) ≤ 3 (good fit); CFI ≥ 0.950 (good fit); Tucker–Lewis Index (TLI) ≥ 0.960 (good fit); RMSEA ≤ 0.060 (good fit) with 90% CI and *p* ≥ 0.050, Standardized Root Mean Residual (SRMR) ≤ .080 (good fit).

##### Item Analysis

The discrimination capacity of the items was determined using the corrected total-element correlation, (*r*_tec_) > 0.200 (Abad et al., 2011) calculated on totals by factor. Furthermore, to know the contribution of each item to reliability, the reliability coefficient per item (*r*_i_) was calculated, expecting values ≥ 0.500 (Fornell and Larcker, 1981).

##### Evidence of Validity Regarding the Relationship With Other Variables

Evidence of convergent, divergent, and discriminant validity was collected. For convergent validity, the AVE of all factors was calculated, with values ≥ 0.500 indicative of convergent validity (Fornell and Larcker, 1981; Cheung and Wang, 2017). Also, convergent validity criterion was considered the factor loadings (λ) ≥ 0.500 (Cheung and Wang, 2017). Finally, the pattern of correlations between the IBCAP-T and PFS-T factors was evaluated, expecting positive or negative correlations as theoretically expected (calculating the attenuation by reliability and controlling the effect of the SDes using partial correlations); Spearman's Rho coefficient was used in this analysis due to the lack of normality (negative Shapiro–Wilk test). For discriminant validity, the r_between−factors_ of each pair of factors of the same scale was calculated, where the values ≤ 0.700 being indicative of discriminant validity (Cheung and Wang, 2017). Also, the $r_{\text{between}-\text{factors}}^{2}$ were compared, indicating discriminant validity as < AVE (Fornell and Larcker, 1981).

##### Reliability Evidence

McDonald's Omega (ω) and greatest lower bound (GLB) coefficients were used because they have been shown to be better estimators of internal consistency than Cronbach's Alpha coefficient (α, Trizano-Hermosilla and Alvarado, 2016). The latter was also calculated because the coefficients, ω and GLB are not yet widely used; therefore, the coefficient, α allows comparison with other works; However, to reduce the impact of non-compliance with the α coefficient assumptions (Batista-Foguet et al., 2004), the 95% confidence interval (CI) is reported. All internal consistency coefficients were calculated from polychoric correlation matrices (Holgado-Tello et al., 2008; Brown, 2015; Desjardins and Bulut, 2018), and the values >0.700 were considered good (George and Mallery, 2017). Finally, in a complementary way, the maximum and minimum split-half reliability was estimated (Abad et al., 2011) interpreting the scores with the same criteria.

##### Norms and Interpretation of Test Scores

As criteria for the interpretability of scores, the following statistics by factor were calculated: mean, standard deviation, skewness and kurtosis coefficients, Shapiro–Wilk test, and SEM.

The programming language, R version 4.0.3 was used with lavaan package (R Core Team, 2020) and the software, SPSS v.24 (IBM Corporation, 2016) and Microsoft Excel Professional Plus 2016 (Microsoft Corporation, 2016) were used for the statistical treatment of the data.

## Results

### Validity Evidence Concerning the Internal Structure of the Instrument

Mardia test indicated no symmetry and kurtosis multivariate indicated both IBCAP-T (symmetry multivariate = 4,106.741, *p* < 0.001; kurtosis multivariate = 22.255, *p*< 0.001) and PFS-T (symmetry multivariate = 2,668.980, *p* < 0.001; kurtosis multivariate = 21.461, *p*< 0.001), for which the ULSM estimation was used. Confirmatory models of each are presented in Table 2.

**Table 2:** Goodness-of-fit indicators of the IBCAP-T and PFS-T confirmatory models with ULSM estimation and polychoric correlation matrix (*N* = 200).

	**χ** ^**2**^	**df**	***p* (** **χ** ^**2**^ **)**	**χ** ^**2**^ **/df**	**CFI**	**TLI**	**RMSEA (CI 90%)**	***p* (RMSEA)**	**SRMR**
**IBCAP-T**									
M1 (30 items)	1,107.976	384	<0.001	2.885	0.968	0.964	0.097 (0.086, 0.109)	<0.001	0.062
M2 (25 items)	9,555.120	275	<0.001	34.746	0.350	0.291	0.412 (0.404, 0.420)	<0.001	0.338
M3 (25 items)	479.541	254	<0.001	1.888	0.984	0.981	0.067 (0.051, 0.083)	0.045	0.049
**PFS-T**									
M4 (49 items)	4,718.315	1,117	<0.001	4.224	0.759	0.747	0.127 (0.123, 0.131)	<0.001	0.127
M5 (25 items)	1,138.266	275	<0.001	4.139	0.888	0.878	0.126 (0.118, 0.134)	<0.001	0.128
M6 (25 items)	469.795	269	<0.001	1.747	0.974	0.971	0.061 (0.049, 0.073)	0.061	0.066

*IBCAP-T, Inventory Brief Child Abuse Potential Translated; PFS-T, Protective Factors Survey Translated; M1, Original 7-factor model; M2, Modified Independent 7-factor model; M3, Modified Correlated 7-factor model; M4, Original 5-factor model; M5, Modified Independent 4-Factor Model; M6, Modified Correlated 4-Factor Model; CFI, Comparative Fit Index; TLI, Tucker–Lewis Index; RMSEA, Root Mean Square Error of Approximation; SRMR, Standardized Root Mean Residual; CI, Confidence Interval; p, p-value*.

Table 2 shows that the IBCAP-T 7-correlated factor model was confirmed by eliminating five items, fitting better than the original model with 30 items and the modified independentable 3 model. In the PFS-T, the NA factor was eliminated, achieving a good fit with a model of 4 correlated factors and 25 items.

The item deletion was performed by the modification indices. These allow decisions for re-specification of the models and reduce the size of the chi-square statistic by removing parameters (Hair et al., 1999; Escobedo-Portillo et al., 2016). Also, an additional criterion to remove items was to present correlated error variances and have a factor loading <0.40. These criteria were considered important because, together, they allow for identifying those items that may not have a relationship with the construct to which they theoretically belong and those items that have an exogenous source of variance (non-random variance unexplained by the construct). This model of re-specification procedure was chosen because it allows for a more parsimonious model to be generated (Brown, 2015). Therefore, the items with high modification indices and factor loadings <0.40 were eliminated one by one until an acceptable fit was reached in the different fit indices.

**Figure 1:**
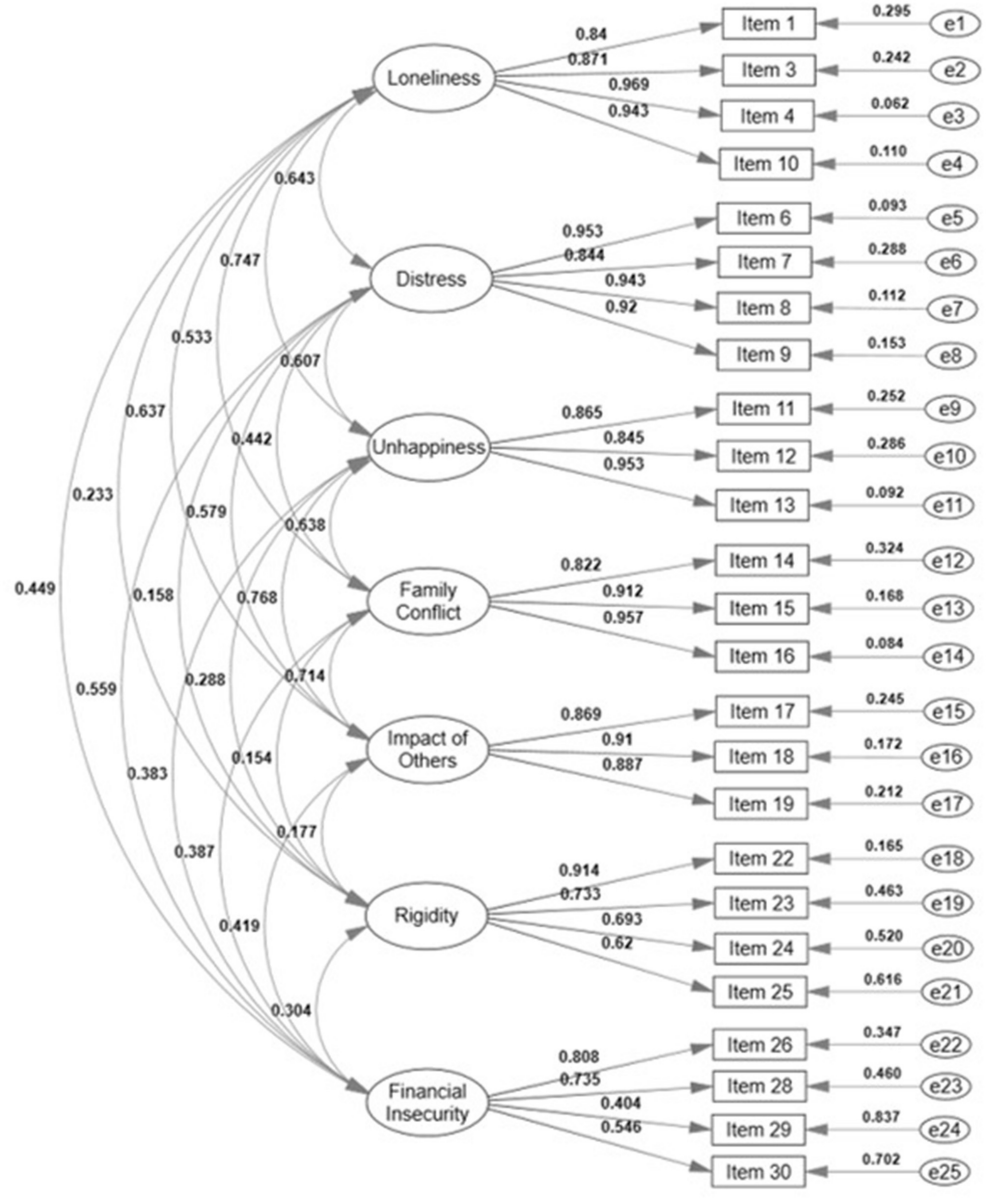
Modified correlated 7-factor model of IBCAP-T. The estimates of the presented factor loadings, variances, and covariances are standardized.

**Figure 2:**
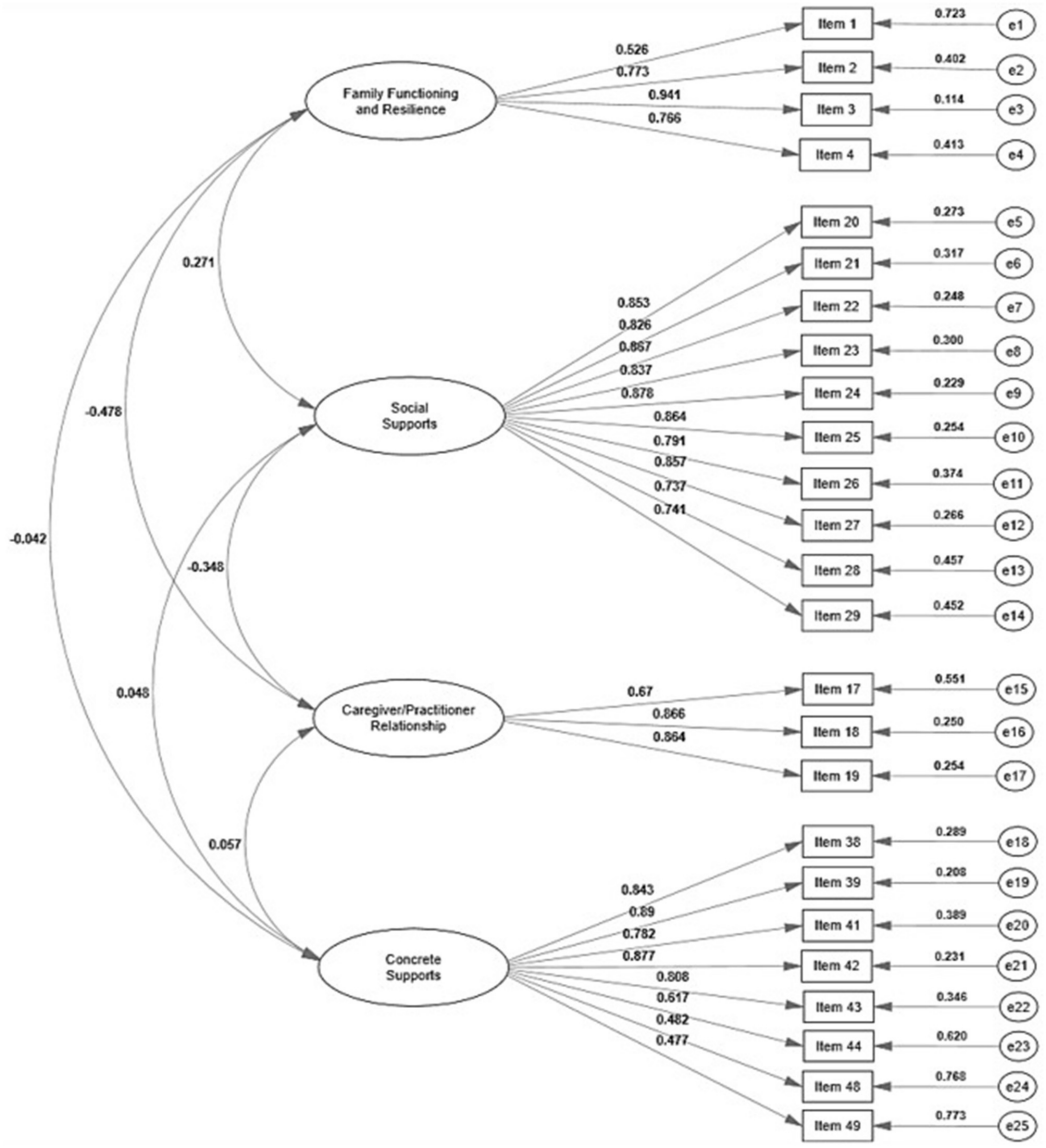
Modified correlated 4-factor model of PFS-T. The estimates of the presented factor loadings, variances, and covariances are standardized.

As can be seen, the contrast of rival models (original vs. modified and correlated vs. modified independent) allows us to safely conclude that the data better fit the theoretical models which include both the elimination of parameters with residuals that covariate with each other (modified models eliminating variables) as a degree of covariation between the factors of the same scale (correlated models). This was true both for IBCAP-T and PFS-T; however, the elimination of NA factor in the PFS-T may indicate a differential functioning of the items in the Mexican culture, in such a way that Nurturing and Attachment are manifested differently from what is found in the context of the United States. It is worth mentioning that the variance explained by the factor should be taken with caution because they are correlated structures in which there may be an overestimation of the variance explained; However, the theoretical background of the IBCAP and the PFS suggests that a structure of correlated factors is the most expected one (Liel et al., 2019; Sprague-Jones et al., 2019). The factorial structures of the models with the best fit of the IBCAP-T and the PFS-T are presented in Figures 1, 2, respectively.

### Item Analysis

In the item analysis, the results for the IBCAP-T and PFS-T are shown in Tables 3, 4.

**Table 3:** Item analysis, reliability evidence, and statistics for the interpretability of the IBCAP-T (*N* = 200).

**Factor**	**Item**	**Item analysis**		**Reliability**					**Interpretability**					
		**r**_**tec**_	**r**_**i**_	**α**_**Ord**_ **(CI 95%)**	**ω**_**Ord**_	**GLB**_**Ord**_	***r*_**min**_**	***r*_**max**_**	**M**	**SD**	**Skew**	**Kurt**	**S–W (2-tailed *p*)**	**SEM**
F1	ITEM1	0.859	0.740	0.949 (0.939, 0.959)	0.950	0.971	0.941	0.954	3.318	1.823	0.460	−0.874	0.928 (< 0.001)	0.310
	ITEM3	0.883	0.783											
	ITEM4	0.943	0.940											
	ITEM10	0.818	0.896											
F2	ITEM6	0.899	0.911	0.954 (0.945, 0.963)	0.954	0.970	0.944	0.970	3.304	1.741	0.406	−0.883	0.941 (< 0.001)	0.302
	ITEM7	0.854	0.746											
	ITEM8	0.874	0.894											
	ITEM9	0.922	0.857											
F3	ITEM11	0.797	0.774	0.917 (0.900, 0.933)	0.920	0.951	0.789	0.791	2.305	1.415	0.767	−0.434	0.844 (< 0.001)	0.313
	ITEM12	0.794	0.747											
	ITEM13	0.906	0.912											
F4	ITEM14	0.763	0.717	0.924 (0.908, 0.938)	0.928	0.941	0.764	0.850	3.067	1.783	0.562	−0.766	0.913 (< 0.001)	0.433
	ITEM15	0.889	0.844											
	ITEM16	0.887	0.919											
F5	ITEM17	0.829	0.780	0.919 (0.902, 0.934)	0.919	0.916	0.813	0.814	3.077	1.782	0.487	−0.855	0.915 (< 0.001)	0.516
	ITEM18	0.831	0.841											
	ITEM19	0.845	0.807											
F6	ITEM22	0.699	0.847	0.832 (0.797, 0.863)	0.835	0.867	0.790	0.867	3.209	1.383	0.185	−0.532	0.969 (< 0.001)	0.504
	ITEM23	0.729	0.613											
	ITEM24	0.567	0.571											
	ITEM25	0.648	0.502											
F7	ITEM26	0.605	0.700	0.699(0.637, 0.755)	0.726	0.764	0.673	0.733	3.969	1.354	−0.188	−0.481	0.983 (0.019)	0.658
	ITEM28	0.619	0.615											
	ITEM29	0.258	0.326											
	ITEM30	0.485	0.438											

*Values that did not meet the defined criteria are highlighted. F1, Loneliness; F2, Distress; F3, Unhappiness; F4, Family Conflict; F5, Impact of Others; F6, Rigidity; F7, Financial Insecurity; r_tec_, Corrected Total-Element Correlation correlating each item with the total of the factor to which it belongs.; r_i_, Reliability by Item following the formula $r_{i}=\frac{\lambda_{i}^{2}}{\lambda_{i}^{2}+Var\left(\varepsilon_{i}\right)},$ where $\lambda_{\text{i}}^{2}$ is the factor loading raised to the square of the i-th item, and Var(ε_i_) is the error variance of the i-th item (Fornell and Larcker, 1981); α_Ord_, Ordinal Cronbach's Alpha Coefficient; CI, Confidence interval; ω_Ord_, Ordinal McDonald's Omega Coefficient; GLB_Ord_, Ordinal Greatest Lower Bound Coefficient; r_min_, minimum split-half reliability; r_max_, maximum split-half reliability; M, Mean; SD, Standard Deviation; Skew, Skewness coefficient; Kurt, Kurtosis coefficient; S–W, Shapiro–Wilk test; p, p-value; SEM, Standard Error of Measurement*.

**Table 4:** Item analysis, reliability evidence, and statistics for the interpretability of the PFS-T (*N* = 200).

**Factor**	**Item**	**Item analysis**		**Reliability**					**Interpretability**					
		***r*_**tec**_**	***r*_**i**_**	**α**_**Ord**_ **(CI 95%)**	**ω**_**Ord**_	**GLB_**Ord**_**	***r*_**min**_**	***r*_**max**_**	**M**	**SD**	**Skew**	**Kurt**	**S–W (2-tailed *p*)**	**SEM**
F1	ITEM1	0.503	0.421	0.838 (0.805, 0.868)	0.847	0.883	0.790	0.876	5.508	1.265	−1.011	0.557	0.907 (<0.001)	0.433
	ITEM2	0.791	0.658											
	ITEM3	0.709	0.892											
	ITEM4	0.693	0.650											
F2	ITEM20	0.829	0.758	0.955 (0.946, 0.963)	0.956	0.975	0.905	0.972	4.924	1.633	−0.784	−0.293	0.919 (<0.001)	0.258
	ITEM21	0.821	0.723											
	ITEM22	0.839	0.778											
	ITEM23	0.816	0.736											
	ITEM24	0.824	0.793											
	ITEM25	0.848	0.773											
	ITEM26	0.773	0.679											
	ITEM27	0.844	0.763											
	ITEM28	0.730	0.617											
	ITEM29	0.722	0.621											
F3	ITEM17	0.673	0.549	0.843 (0.811, 0.872)	0.850	0.886	0.722	0.813	3.205	1.616	0.324	−0.865	0.949 (<0.001)	0.546
	ITEM18	0.795	0.776											
	ITEM19	0.662	0.773											
F4	ITEM38	0.784	0.745	0.896 (0.875, 0.915)	0.902	0.922	0.818	0.931	1.458	1.240	1.399	2.134	0.878 (<0.001)	0.346
	ITEM39	0.825	0.811											
	ITEM41	0.734	0.668											
	ITEM42	0.815	0.792											
	ITEM43	0.744	0.700											
	ITEM44	0.583	0.499											
	ITEM48	0.482	0.386											
	ITEM49	0.473	0.382											

*Values that did not meet the defined criteria are highlighted. F1, Family Functioning and Resilience; F2, Social Supports; F3, Caregiver/Practitioner Relationship; F4, Concrete Supports; r_tec_, Corrected Total-Element Correlation correlating each item with the total of the factor to which it belongs.; r_i_,Reliability by Item following the formula $r_{i}=\frac{\lambda_{i}^{2}}{\lambda_{i}^{2}+Var\left(\varepsilon_{i}\right)}$ where $\lambda_{\text{i}}^{2}$ is the factor loading raised to the square of the i-th item, and Var(ε_i_) is the error variance of the i-th item (Fornell and Larcker, 1981); α_Ord_, Ordinal Cronbach's Alpha Coefficient; CI, Confidence interval; ω_Ord_, Ordinal McDonald's Omega Coefficient; GLB_Ord_, Ordinal Greatest Lower Bound Coefficient; r_min_, minimum split-half reliability; r_max_, maximum split-half reliability; M, Mean; SD, Standard Deviation; Skew, Skewness coefficient; Kurt, Kurtosis coefficient; S–W, Shapiro–Wilk test; p, p value; SEM, Standard Error of Measurement*.

Table 3 shows that the IBCAP-T items had discrimination levels that ranged between 0.258 and 0.943, and reliability levels between 0.326 and 0.944. In the PFS-T items, discrimination ranged between 0.473 and 0.848 and reliability ranged between 0.382 and 892 (Table 4). In both instruments, the levels of discrimination and reliability were good or excellent. For a list of items of both psychometric instruments in English and Spanish, see Appendix D.

### Evidence of Validity Regarding the Relationship With Other Variables

Evidence of convergent validity (λ and AVE of the M3 and M6 models) and discriminants (*r*_bf_ and $r_{\text{bf}}^{2}$) are presented in Figures 1, 2, and in Table 5 for the IBCAP-T and PFS-T, respectively. Also, the correlations between the IBCAP-T and PFS-T factors (convergent and divergent validity) are presented in Table 6.

**Table 5:** Evidence of convergent and discriminant validity of the IBCAP-T and PFS-T (*N* = 200).

	**IBCAP-T**								**PFS-T**			
	**1**	**2**	**3**	**4**	**5**	**6**	**7**		**1**	**2**	**3**	**4**
1 L	–	0.413	0.558	0.284	0.406	0.054	0.202	1 FFR	–	0.073	0.228	0.002
2 D	0.643	–	0.368	0.195	0.335	0.025	0.312	2 SS	0.271	–	0.121	0.002
3 U	0.747	0.607	–	0.407	0.590	0.083	0.147	3 CPR	−0.478	−0.348	–	0.003
4 FC	0.533	0.442	0.638	–	0.510	0.024	0.150	4 CS	−0.042	0.048	0.057	–
5 IO	0.637	0.579	0.768	0.714	–	0.031	0.176	AVE	0.587	0.683	0.648	0.547
6 R	0.233	0.158	0.288	0.154	0.177	–	0.092					
7 FI	0.449	0.559	0.383	0.387	0.419	0.304	–					
AVE	0.897	0.908	0.876	0.887	0.876	0.683	0.523					

*Values that did not mee the defined criteria are highlighted. The correlation between factors (r_bf_) is below the diagonal. The coefficient of determination between factors ($r_{\text{bf}}^{2}$) is above the diagonal. AVE, Average variance extracted*.

**Table 6:** Evidence of convergent and divergent validity between the IBCAP-T and PFS-T factors adjusted for reliability and bias for Social Desirability (*N* = 200)^a^^,^^b^.

	**1 IBCAP-T**	**2 IBCAP-T**	**3 IBCAP-T**	**4 IBCAP-T**	**5 IBCAP-T**	**6 IBCAP-T**	**7 IBCAP-T**
**Crude correlations** ^**c**^							
1 PFS-T	−0.442^**^	−0.235^**^	−0.351^**^	−0.537^**^	−0.365^**^	−0.042	−0.170^*^
2 PFS-T	−0.349^**^	−0.216^**^	−0.261^**^	−0.261^**^	−0.228^**^	−0.035	−0.085
3 PFS-T	0.595^**^	0.385^**^	0.501^**^	0.381^**^	0.422^**^	0.298^**^	0.266^**^
4 PFS-T	0.075	0.174^*^	0.156^*^	0.153^*^	0.109	0.026	0.377^**^
**Reliability-adjusted correlations** ^**d**^							
1 PFS-T	−0.477^**^	−0.254^**^	−0.383^**^	−0.589^**^	−0.406^**^	−0.048	−0.207^**^
2 PFS-T	−0.359^**^	−0.222^**^	−0.271^**^	−0.272^**^	−0.241^**^	−0.038	−0.098
3 PFS-T	0.641^**^	0.415^**^	0.546^**^	0.417^**^	0.468^**^	0.340^**^	0.323^**^
4 PFS-T	0.079	0.184^**^	0.167^*^	0.164^*^	0.119	0.029	0.449^**^
**Correlations bias by Social Desirability** ^**e**^ ^**,**^ ^**f**^							
1 PFS-T	−0.446^**^	−0.154^*^	−0.329^**^	**–0.561** ^**^	−0.345^**^	−0.015	–0.111
2 PFS-T	−0.308^**^	−0.204^**^	−0.207^**^	−0.255^**^	−0.213^**^	−0.010	−0.096
3 PFS-T	0.546^**^	0.309^**^	0.434^**^	0.364^**^	0.383^**^	**0.309** ^**^	0.246^**^
4 PFS-T	−0.014	0.128	0.100	0.129	0.062	0.004	**0.449** ^**^
**Attenuation index** ^**g**^							
1 PFS-T	7.34	7.48	8.36	8.83	10.10	12.50	17.87
2 PFS-T	2.79	2.70	3.69	4.04	5.39	7.89	13.27
3 PFS-T	7.18	7.23	8.24	8.63	9.83	12.35	17.65
4 PFS-T	5.06	5.43	6.59	6.71	8.40	10.34	16.04
**Difference by bias** ^**h**^							
1 PFS-T	−0.031	−0.100	−0.054	−0.028	−0.061	−0.033	−0.096
2 PFS-T	−0.051	−0.018	−0.064	−0.017	−0.028	−0.028	−0.002
3 PFS-T	0.095	0.106	0.112	0.053	0.085	0.031	0.077
4 PFS-T	0.093	0.056	0.067	0.035	0.057	0.025	0.000

a *The values of the correlations after being corrected for reliability and bias of social desirability and passed from p < 0.05 to p > 0.05 are underlined*.

b *Correlations that increased after removing the effect of social desirability were marked in bold*.

c *Spearman's Rho coefficients were calculated due to the absence of univariate normality in the total scores by factor*.

d *The correlations were adjusted with the formula, $r_{true}=\frac{r_{observed}}{\sqrt[{2}]{r_{xx}.r_{yy}}}$ where r_xx_ y r_yy_ are the GLB_Ord_ coefficients by factor, in such a way that the adjusted correlation is an estimate of the true correlation*.

e *The partial correlations were worked with the factors of Self-deception and Printing Handling in such a way that the reported partial correlations are of the second order*.

f *The bias was conducted on the correlations previously corrected by reliability, using in all cases the coefficient GLB_Ord_ to make the adjustment*.

g $Attenuation\;index\;=\left[(r_{true}-r_{observed})/r_{true}\right]\;(100)$.

h $Difference\;by\;bias=r_{true}-r_{biased}$.

* *p < 0.05*.

** *p < 0.01*.

It can be seen in Figures 1, 2 that the λ meet the criteria (λ > 0.50) to assume convergent validity for both instruments (except 1 item from the IBCAP-T and 2 items from the PFS-T). Since the factor loadings are the correlation of the item with its latent factor, it is expected that higher values in λ items indicate convergent validity. Meanwhile, the AVE indicates the amount of variance explained by the construct such that the higher the AVE, the more it is argued that the items contribute to the measurement, i.e., high AVE values indicate the convergence of the items of a construct. In this regard, the AVE show that both for the IBCAP-T and the PFS-T, all factors showed an explained variance <0.50 (see Table 5).

In terms of discriminant validity, the correlations between factors (*r*_bf_), of the same scale indicates the absence of collinearity, that is, the items of one factor measure the same as the items of a different factor. For this reason, although it is expected that there is a low or medium correlation between the factors that make up a scale, it is expected that these correlations do not reach a value high enough to cause confusion in the dimensions of the construct. In the same sense, the Squared correlation between factors ($r_{\text{bf}}^{2}$) can be understood as the shared variance between the factors of the same scale, that is, between the dimensions of a construct. Thereupon, it is expected that the items of the same factor shared more variance with each other (AVE) than that they share with another factor ($r_{\text{bf}}^{2}$), so values of $r_{\text{bf}}^{2}$ must be less than the values of AVE to assert discriminant validity. It can be seen in Table 5 for the IBCAP-T, that only three of the 21 *r*_bf_ are slightly above 0.700 (see values below the diagonal marked with -); However, when comparing the $r_{\text{bf}}^{2}$ (observe the values above the diagonal marked with -) and the AVE, in each comparison, the AVE values are greater than the $r_{\text{bf}}^{2}$, which indicates that the variance shared by the items of the same factor is greater than the shared variance between factors. In the PFS-T, all the discriminant validity indicators met the expected criteria.

Regarding the correlations between the IBCAP-T factors and the PFS-T factors, Table 6 shows that the crude correlations adjusted for reliability increased in a range that goes from 2.70 to 17.89%, which can be interpreted as the percentage of the true correlation that is not registered due to the measurement error. On the other hand, the bias by SD showed, in most of the correlations, lower values than the crude correlations, which represents a high impact of the SD. In terms of convergent and divergent validity, median correlations were found with *p*-values < 0.05 and 0.01 even after removing the effect of social desirability, although factor 4 of the PFS-T only moderately correlated with factor 7 of the IBCAP- T. In the same sense, factor 6 of the IBCAP-T only moderately correlated with factor 3 of the PFS-T.

### Reliability Evidence

Tables 3, 4 show the reliability coefficients by factor. It is notable that the only coefficient that did not obtain a value ≥ 0.700 was the α coefficient in factor 7 of the IBCAP-T. On the other hand, both in the IBCAP-T and PFS-T, the relationship α ≤ ω ≤ GLB was maintained.

### Norms and Interpretation of Tests Scores

Tables 3, 4 also show that no factor had measures normally distributed. In the IBCAP-T, all the averages were <4 with SD close to 1, while the PFS-T showed means >4 in the FFR and SS factors, and <4 in the CPR and CS factor, the latter having the lower mean (M) and SD (M = 1,458, SD = 1,240). Finally, it is observed that the factor with the highest SEM was the FI factor of the IBCAP-T, in contrast to the SS factor of the PFS-T that showed the lowest SEM.

## Discussion

The main purpose of the present study was to collect evidence of validity and reliability of the IBCAP and PFS in versions translated into Spanish. The results showed that both instruments have adequate psychometric properties.

By doing factor analysis and estimating reliability from polychoric correlation matrices, more refined and robust results were achieved that better reflect the psychometric characteristics of the instruments (Holgado-Tello et al., 2006, 2007, 2008; Brown, 2015; Desjardins and Bulut, 2018). In addition, the collection of different validity indicators and their consistency is a better approximation to reality than those approaches focused on a single indicator, because each of the analysis, estimation method, and psychometric indicator has limitations or even biases that make a complementary approach necessary which allows for a triangulation of results (Kimchi et al., 1991; Shadish, 1993; Letourneau and Allen, 1999; Heale and Forbes, 2013).

The IBCAP-T was the instrument that required the least adjustments to achieve a satisfactory model, since only five items were eliminated but the structure of seven factors was maintained, which are congruent with the factors of the original extended version of Milner (1986) as well as with the short versions of Ondersma et al. (2005), Ellonen et al. (2019) and Liel et al. (2019). It is noteworthy that the CFI and TLI were adequate with the initial 30 items; however, the RMSEA showed values outside the acceptable in the original model, probably because this indicator is sensitive to the number of estimated parameters and sample size (Kline, 2011).

At the item level, in the IBCAP-T, the levels of discrimination and reliability evidenced the potential for a classificatory use of the instrument, given that most of the items adequately differentiate between subjects with high and low true scores, and all of the Items contribute significantly to reliability.

In terms of validity, the IBCAP-T successfully met all indicators, being a measure that provides valid test scores even after considering the effect of social desirability. Likewise, the correlations between the factors of the IBCAP-T and the PFS-T were congruent with what was expected, since negative (divergent) correlations were found with the factors of SS and FFR, and positive (convergent) with CPR and CS, although the latter only had a medium relationship with the FI factor. This lack of relation of the IBCAP-T factors with the may be due, in part, to the effect of social desirability on the responses of the subjects; that is, the respondents have a way of answering which tend to be self-positive descriptions, such that their responses are consistently different from their true values (Mikulic et al., 2016).

In the PFS-T, the 5-factor model of Sprague-Jones et al. (2019) was not confirmed and the 4-factor model that was confirmed does not coincide in content with that reported by the FRIENDS National Center (FRIENDS National Center for Community Based Child Abuse Prevention, 2018) since the CPR factor that was confirmed in this study is only found in the version by Sprague-Jones et al. (2019). It should be noted that this factor is named for its use in the United States in abuse prevention programs; however, in this study, the factor can be better interpreted if it is considered a measure of relationship with others in general. The fact that the original 5-factor model was not confirmed can be partially explained by the different changes that were made in the scale, both in the response options (all items were unified on a scale from 1 to 7) and in the disaggregation of some items (see Appendix A), aspect that can also explain the elimination of 24 items.

Despite the modifications made to the PFS-T, the items of the adjusted 4-factor model showed adequate levels of discrimination and reliability. The same is true at the level of factors for internal consistency and validity of the different indicators. However, in the correlations with the IBCAP-T factors, the CPR factor was the only one that correlated with the Rigidity factor. It is noteworthy that the CPR factor showed the highest correlations with the IBCAP-T factor but also showed the greatest effects on social desirability, since it consistently showed the highest levels of difference between the estimated true correlation and the correlation controlled by SD.

On the interpretability, the report of the SEM is important for estimating intervalar true scores of an individual (Gempp, 2006), which is an important aspect for the use of the instrument in individual diagnoses. In this sense, the extension of the instruments ratifies the practical potential of the instruments in individual evaluation, since being long instruments (more than 20 questions), the effects of the SEM in decisions at the individual level are mitigated (e.g., correctly conclude the presence of risk factors in an individual; being able to detect medium effects in before–after comparisons and not just large effects) (Sijtsma, 2011).

It is important to note that the IBCAP-T showed a more robust behavior with respect to previous validation studies since the modifications made to the Spanish version were minor, achieving comparability with other existing versions. This does not happen with the PFS-T because substantial changes were introduced to the adaptation to the Mexican population. A direct consequence of the lack of robustness of PFS-T is the inability to make comparisons with other versions of the instrument. However, the numerous validity and reliability evidence obtained, as well as the statistics for the interpretability obtained in this study indicate that the use of the PFS-T in the Mexican population is extremely promising in terms of being able to have indices of validity, reliability, and feasibility, which are unprecedented in Mexico and which will allow investigations into child abuse area.

This study has some limitations. It is important to explore semantic aspects that may affect the quality of the items and that could have been omitted due to the lack of a back-translation process. However, this aspect is cushioned by the contribution of three experts, one on linguistic issues, another on expertise on the subject, and a third in the development of psychometric instruments. In terms of the heterogeneity and sample size, it is necessary to carry out subsequent studies that analyze, in larger samples, the differential functioning of the instruments mainly in variables, such as sex, family structure, and the preference of children. However, the intrinsic complexity of child abuse, the territorial extent, and the cultural diversity of the country always demand a careful use of these instruments in the Mexican territory, contemplating variables, such as the region (north, center, or south) and socioeconomic conditions, as well as the inclusion of indigenous communities. The analysis of all these variables is beyond the scope of this study; however, valuable information is provided on the usability of the two tools. On the other hand, when making adjustments to the factorial structure after checking the modification indices (and therefore make apparently exploratory use of the CFA), there is a risk of biases due to “chance capitalization” (Batista-Foguet et al., 2004); However, given the severe defects of the EFA (Batista-Foguet et al., 2004), and the strengths of the Confirmatory Factor Analysis (CFA) (Brown, 2015), the process of modifying the models by eliminating items using the CFA is highly preferable to the use of the EFA, despite the probable chance capitalization. Furthermore, the re-specification of the models by eliminating the items with correlated error variances is a process that generates alternative models that have a legitimate psychometric interpretation, in contrast to the process of “correlating the error variance of the parameters” which lacks psychometric interpretation, despite its relatively extended use. Although, in general terms, re-specification can be considered a form of exploration, the conditions of its development are considerably different because the re-specification that we carry out in this work started from a pre-existing theoretical model that was gradually simplified (more parcimonious models) and that it is interpretable within the framework of general theories that contain it (child abuse theories); There were no cross-loads (greater restriction in the specification compared to the EFA) and it was constantly possible to contrast the fit of re-specified models, which allowed to achieve solidly integrated and psychometrically interpretable factorial structures. Despite all of the above, for further development, we intend to strengthen the inferences made from the results obtained in this work, checking the model in different and larger samples. A fourth limitation lies in the size of the sample and distribution by sex and children; we did not conduct an analysis of invariance measurement, so that comparisons between subgroups are inadvisable until we have sufficient information (Chen, 2007). The fifth limitation lies in that, although interpretability indicators were reported, it would be interesting to conduct a study to establish non-arbitrary cut points (Abad et al., 2011).

## Conclusion

This study sought to gather solid evidence on the validity and reliability of the Spanish translated versions of the IBCAP-T and PFS-T. One solid starting point was provided for the development of tools to determine a valid and reliable way, the presence or absence of factors that may increase the likelihood of child abuse as well as those factors that can reduce its incidence.

## Data Availability Statement

The raw data supporting the conclusions of this article will be made available by the authors, without undue reservation.

## Ethics Statement

Ethical approval was not provided for this study on human participants because Informed consent was provided by all participants. This study passed the approval of the Master's and Doctorate Program in Psychology Committee at Universidad Nacional Autónoma de México (UNAM). The patients/participants provided their written informed consent to participate in this study.

## Author Contributions

AS-M and AA supported, planned, and developed the research. EC and PA supervised and advised data collection. AS-M performed the ordering, statistical analysis of the data, and wrote the first draft. SC-M and SS-C supervised the statistical analysis of data, revised, and edited the manuscript. All authors contributed to the article and approved the submitted version.

## Conflict of Interest

The authors declare that the research was conducted in the absence of any commercial or financial relationships that could be construed as a potential conflict of interest.

## Publisher's Note

All claims expressed in this article are solely those of the authors and do not necessarily represent those of their affiliated organizations, or those of the publisher, the editors and the reviewers. Any product that may be evaluated in this article, or claim that may be made by its manufacturer, is not guaranteed or endorsed by the publisher.
