# Supplementary material for: Spanish Adaptation of the Inventory Brief Child Abuse Potential and the Protective Factors Survey
Source: Front Psychol. 2021 Aug 3;12:705228. doi: 10.3389/fpsyg.2021.705228 (PMC8368729; doi:10.3389/fpsyg.2021.705228)
Supplement: Supplementary file 1 [file Data_Sheet_1.PDF]

Supplementary Material

Appendix A. List of changes in items based on the evaluation of translations for the 21 items of the IBCAP and the 29 items of the PFS

Evaluation	# Items		
	IBCAP	PFS	Total
Translation 1 is better	8	1	9
Translation 2 is better	3	12	15
Translation 3 is better	6	12	18
Translations 1 and 2 are better	0	1	1
Translations 1 and 3 are better	2	1	3
Translations 2 and 3 are better	0	2	2
All three translations are just as good	2	0	2
Total	21	29	50

Inventory Brief Child Abuse Potential (IBCAP)

#	Original version	Translation	Preliminary version
1	1. I often feel alone.	Seguido me siento solo.	R1. Seguido me siento solo. **L**
2	2. Sometimes I feel all alone in the world.	A veces me siento solo en el mundo.	R2. A veces me siento solo en el mundo. **L**
3	3. I often feel lonely inside.	Seguido me siento solo por dentro.	R3. Seguido me siento solo por dentro. **L**
4	4. I am often depressed.	Seguido estoy deprimido.	R10. Seguido estoy deprimido. **L**
5	5. I often feel very alone.	Seguido me siento muy solo.	R4. Seguido me siento muy solo. **L**
6	6. I often feel very upset.	Seguido me siento muy enojado.	R6. Seguido me siento muy enojado. **D**
7	7. I often feel worthless.	Seguido siento que no sirvo.	R5. Seguido siento que no sirvo. **L**
8	8. I am often upset.	Seguido estoy enojado.	R9. Seguido estoy enojado. **D**
9	9. I am easily upset by my problems.	Me enojo fácilmente por mis problemas.	R7. Me enojo fácilmente por mis problemas. **D**
10	10. People have caused me a lot of pain.	Las personas me han causado mucho dolor.	R17. Las personas me han causado mucho dolor. **IO**
11	11. Other people have made my life hard.	Otras personas me han hecho la vida difícil.	R18. Otras personas me han hecho la vida difícil. **IO**
12	12. Other people have made my life unhappy.	Otras personas han hecho mi vida infeliz.	R19. Otras personas han hecho mi vida infeliz. **IO**
13	13. My family has many problems.	Mi familia tiene muchos problemas.	R14. Mi familia tiene muchos problemas. **FC**
14	14. My family has problems getting along.	Mi familia tiene problemas para llevarse bien.	R15. Mi familia tiene problemas para llevarse bien. **FC**
15	15. My family fights a lot.	Mi familia pelea mucho.	R16. Mi familia pelea mucho. **FC**
16	16. I sometimes worry that my needs will not be met.	A veces me preocupa que mis necesidades no se cumplan.	R21. A veces me preocupa que mis necesidades no se cumplan. **FC**
17	17. Children should be quiet and listen.	Los niños deben de guardar silencio y escuchar.	R22. Los niños deben de guardar silencio y escuchar. **R**
18	18. Children should never disobey.	Los niños nunca deben desobedecer.	R23. Los niños nunca deben desobedecer. **R**
19	19. Everything in a home should always be in its place.	En casa todo debe de estar en su lugar.	R24. En casa todo debe de estar en su lugar. **R**
20	20. I sometimes worry that I will not have enough to eat.	A veces me preocupa no tener suficiente para comer.	R26. A veces me preocupa no tener suficiente para comer. **IF**
21	21. A child needs very strict rules.	Un niño necesita reglas muy estrictas.	R25. Un niño necesita reglas muy estrictas. **R**
22	8. I am often upset and I don’t know why.[a]	Seguido estoy enojado y no sé por qué. [b]	R8. Seguido estoy enojado y no sé por qué. **D**
23	11. My life is good.[a]	Mi vida es buena.[bc]	R11. Mi vida es mala. **U**
24	12. I am a happy person.[a]	Soy una persona feliz.[bc]	R12. Soy una persona infeliz. **U**
25	13. My life is happy.[a]	Mi vida es feliz.[bc]	R13. Mi vida es infeliz. **U**
26	18. I often feel that my needs are not met.[a]	Seguido siento que no se cumplen mis necesidades. [b]	R20. Seguido siento que no se cumplen mis necesidades. **IO**
27	--	--	R27. A veces me preocupa no tener para pagar mis deudas. **FI** [d]
28	--	--	R28. Me preocupa no tener un buen ingreso económico. **FI** [d]
29	--	--	R29. A veces gasto más dinero de lo que debería. **FI** [d]
30	--	--	R30. Siento que tengo un ingreso económico bajo. **FI** [d]

Protective Factors Survey (PFS)

#	Original version	Translation	Preliminary version
1	8. The future looks good for our family.	El futuro depara cosas buenas para mí y mi familia.	D1. El futuro depara cosas buenas para mí y mi familia. **FFR**
2	9. The way my family works together helps us deal with the hard times.	La forma en que mi familia trabaja junta nos ayuda a lidiar con los tiempos difíciles.	D2. La forma en que mi familia trabaja junta nos ayuda a lidiar con los tiempos difíciles. **FFR**
3	10. In my family, we take time to listen to each other.	En mi familia nos tomamos el tiempo de escucharnos.	D3. En mi familia nos tomamos el tiempo de escucharnos. **FFR**

4	11. There are things we do as a family that are special just to us.	Hay actividades que son importantes solo para mi familia y yo	D4. Hay actividades que son importantes solo para mi familia y yo. **FFR**
5	25. It's hard for me to ask for help when I need it.	Es difícil para mí pedir ayuda cuando la necesito.	D5. Es difícil para mí pedir ayuda cuando la necesito. **NA**
6	26. Sometimes I don't know what to do as a parent because of how I was raised.	A veces no sé qué hacer como padre debido a cómo me criaron.	D6. No sabría o no sé qué hacer como padre/madre debido a cómo me criaron. **NA** [e]
7	27. Being a parent is harder than I thought it would be.	Ser padre es más difícil de lo que me imaginé.	D7. Pienso que ser padre es más difícil de lo que me imagino (o imaginé). **NA** [e]
8	13. I feel like I'm always telling my kids "no" or "stop."	Siento que siempre estoy diciéndoles "no hagas esto" a mis hijos.	D8. Siento que siempre estaría o estoy diciéndoles "no hagas esto" a mis hijos. **NA** [e]
9	14. I have frequent power struggles with my kids.	Mis hijos suelen cuestionar mucho mi autoridad.	D9. Siento que los hijos suelen cuestionar mucho la autoridad. **NA** [e]
10	12. My child misbehaves just to upset me.	Mi hijo se porta mal solo para molestarme.	D10. Los hijos se portan mal sólo para molestar a los padres. **NA** [e]
11	15. How I respond to my child depends on how I'm feeling.	Cómo respondo a mi hijo depende de cómo me siento.	D11. Los padres responden a sus hijos dependiendo de cómo ellos mismos se sienten. **NA** [e]
12	1. I have friends on social media/online I can rely on for support.	Tengo amigos en las redes sociales que me apoyan.	D12. Tengo amigos en las redes sociales que me apoyan. **SS**
13	2. I have people who believe in me.	Hay gente que cree en mí.	D13. Hay gente que cree en mí. **SS**
14	3. I have someone in my life who gives me advice, even when it's hard to hear.	Tengo a alguien en mi vida que me da consejos, incluso cuando es difícil de escuchar.	D14. Tengo a alguien en mi vida que me da consejos, incluso cuando es difícil de escuchar. **SS**
15	4. When I am trying to work on achieving a goal, I have friends who will support me.	Cuando intento trabajar para lograr un objetivo, tengo amigos que me apoyarán.	D16. Cuando intento trabajar para lograr un objetivo, tengo amigos que me apoyan. **SS** [f]
16	5. When I need someone to look after my kids on short notice, I can find someone I trust.	Cuándo necesito que alguien cuide a mis hijos puedo decirle a alguien, incluso con poca anticipación.	D15. Si necesitara que alguien cuide a mis hijos cuento con personas para decirles, incluso con poca anticipación. **SS** [e]
17	6. I have people I trust to ask for advice about:	Tengo personas en las que confío para pedir consejos sobre:	--
	a. Money/Bills/Budgeting	a. Dinero/Facturas/Presupuestos	D20. Tengo personas en las que confío para pedir consejos sobre: [Dinero/Facturas/Presupuestos] **SS** [g]
	b. Relationships and/or My Love Life	b. Relaciones y/o mi vida amorosa	D21. Tengo personas en las que confío para pedir consejos sobre: [Relaciones y/o mi vida amorosa] **SS** [g]
	c. Food/Nutrition	c. Alimentación/Nutrición	D22. Tengo personas en las que confío para pedir consejos sobre: [Alimentación/Nutrición] **SS** [g]
	d. Stress, Anxiety, and/or Depression	d. Estrés, ansiedad o depresión.	D23. Tengo personas en las que confío para pedir consejos sobre: [Estrés, ansiedad o depresión.] **SS** [g]
	e. Parenting/My Kids	e. Paternidad / Mis Niños	D24. Tengo personas en las que confío para pedir consejos sobre: [Paternidad / Hijos] **SS** [g]
	f. None of the above	f. Ninguna de las anteriores [k]	--
18	7. I have people I trust to ask for advice about:	Tengo personas en las que confío para pedir consejos sobre:	--
	a. Work	a. Trabajo	D25. Tengo personas en las que confío para pedir consejos sobre: [Trabajo] **SS** [h]
	b. Housing and/or Emergency Shelter	b. Alojamiento y/o refugio de emergencia	D26. Tengo personas en las que confío para pedir consejos sobre: [Alojamiento y/o refugio de emergencia] **SS** [h]
	c. Education	c. Educación	D27. Tengo personas en las que confío para pedir consejos sobre: [Educación] **SS** [h]
	d. Legal Issues	d. Asuntos legales	D28. Tengo personas en las que confío para pedir consejos sobre: [Asuntos legales] **SS** [h]
	e. Medical/Dental Care	e. Atención médica / dental	D29. Tengo personas en las que confío para pedir consejos sobre: [Atención médica / dental] **SS** [h]
	f. None of the above	f. Ninguna de las anteriores [k]	--
19	28. There never seems to be enough money to buy something or go somewhere just for fun.	Nunca hay suficiente dinero para comprar algo. Nunca hay suficiente dinero para ir a algún lugar sólo por diversión.	D30. Nunca hay suficiente dinero para comprar algo. **CS** D31. Nunca hay suficiente dinero para ir a algún lugar sólo por diversión. **CS**
20	18. Within the past year, we worried whether our food would run out before we got money to buy more.	El año pasado nos preocupamos por que la comida se acabara antes de que tuviéramos suficiente dinero para comprar más.	D32. El año pasado nos preocupamos por que la comida se acabara antes de que tuviéramos suficiente dinero para comprar más. **CS**
21	19. Within the past year, the food we bought just didn't last and we didn't have money to get more.	Durante el año pasado, la comida que compramos simplemente no duró y no teníamos dinero para obtener más.	D33. Durante el año pasado, la comida que compramos simplemente no duró y no teníamos dinero para obtener más. **CS**
22	17. I am able to afford the food I want to feed my family.	Puedo comprar suficiente comida para alimentar a mi familia.	D34. Puedo comprar suficiente comida para alimentar a mi familia. **CS**
23	29. I worry about money.	Me preocupa el dinero.	D35. Me preocupa el dinero. **CS**
24	16. I have trouble affording what I need each month.	Tengo problemas para pagar lo que necesito cada mes.	D36. Tengo problemas para pagar lo que necesito cada mes. **CS**
25	20. In the past month, were you unable to pay for...	El mes pasado te fue imposible pagar:	--
	a. Rent or mortgage	a. La renta/hipoteca	D37. En los seis meses pasados me fue difícil pagar: [La renta/hipoteca] **CS** [i]

	b. Utilities or bills (electricity/gas/heat, etc.)	b. Las cuentas (la luz, el agua, el gas)	D38. En los seis meses pasados me fue difícil pagar: [Las cuentas (la luz, el agua, el gas)] **CS** [i]
	c. Groceries/food (including baby formula)	c. Comida	D39. En los seis meses pasados me fue difícil pagar: [Comida] **CS** [i]
	d. Child care/daycare	d. La guardería	D40. En los seis meses pasados me fue difícil pagar: [La guardería] **CS** [i]
	e. Medicine, medical expenses, or co-pays	e. Medicinas, gastos médicos	D41. En los seis meses pasados me fue difícil pagar: [Medicinas, gastos médicos] **CS** [i]
	f. Basic household or personal hygiene items	f. Artículos básicos de higiene personal o del hogar.	D42. En los seis meses pasados me fue difícil pagar: [Artículos básicos de higiene personal o del hogar.] **CS** [i]
	g. Transportation (including gas, bus passes, shared rides)	g. Transporte (incluida gasolina, pases de autobús, viajes compartidos)	D43. En los seis meses pasados me fue difícil pagar: [Transporte (incluida gasolina, pases de autobús, viajes)] **CS** [i]
	h. None of the above	h. Ninguna de las anteriores [k]	--
	21. In the past year, were you unable to pay for…	El año pasado porque te fue imposible pagar te paso alguna de las siguientes situaciones	--
	a. Delayed or not gotten medical or dental care	a. Retrasar la atención médica o dental hasta tener dinero	D44. El año pasado, por falta de dinero, me pasó alguna de las siguientes situaciones: [Retrasar la atención médica o dental hasta tener dinero.] **CS** [j]
	b. Been evicted from your home or apartment	b. Te sacaron de tu departamento	D45. El año pasado, por falta de dinero, me pasó alguna de las siguientes situaciones: [Me sacaron de mi departamento/casa.] **CS** [j]
26	c. Lived at a shelter, in a hotel/motel, in an abandoned building, or a vehicle	c. Viviste en un refugio, hotel/motel, edificio abandonado	D46. El año pasado, por falta de dinero, me pasó alguna de las siguientes situaciones: [Viví en un refugio, hotel/motel o edificio abandonado.] **CS** [j]
	d. Moved in with other people, even temporarily, because you could not afford to pay rent, mortgage, or bills	d. Te mudaste con alguien, incluso temporalmente, porque no podías pagar la renta o las cuentas	D47. El año pasado, por falta de dinero, me pasó alguna de las siguientes situaciones: [Me mudé con alguien, incluso temporalmente, porque no podía pagar la renta o las cuentas.] **CS** [j]
	e. Lost access to your regular transportation (e.g. vehicle totaled or repossessed)	e. Perdiste acceso a tu vehículo	D48. El año pasado, por falta de dinero, me pasó alguna de las siguientes situaciones: [Perdí acceso a mi vehículo.] **CS** [j]
	f. Been unemployed when you really needed and wanted a job	f. Estuviste desempleado cuándo querías trabajar	D49. El año pasado, por falta de dinero, me pasó alguna de las siguientes situaciones: [Estuve desempleado cuándo quería trabajar.] **CS** [j]
	g. None of the above	g. Ninguna de las anteriores [k]	--
27	24. When I talk to people about my problems, they just don't seem to understand.	Cuando hablo con la gente sobre mis problemas, parece que no me entienden.	D17. Cuando hablo con la gente sobre mis problemas, parece que no me entienden. **CPR**
28	22. Sometimes I feel like no one understands me.	A veces siento que nadie me entiende.	D18. A veces siento que nadie me entiende. **CPR**
29	23. No one seems to believe that I can change.	Nadie cree que pueda cambiar.	D19. Nadie cree que yo pueda cambiar. **CPR** [f]

Note. PFS = Protective Factors Survey; IBCAP = Inventory Brief Child Abuse Potential; FFR = Family Functioning and Resilience; NA = Nurturing and Attachment; SS = Social Supports; CS = Concrete Supports; CPR = Caregiver-Professional Relationship; L = Loneliness; D = Distress; U = Unhappiness; FC = Family Conflict; IO = Impact of Others; R = Rigidity; FI = Financial Insecurity.

[a] The item belongs to the version of Liel et al. (2019) and was added after the translation process to be able to integrate both versions and have the Unhappiness factor, as well as to be able to separate the Loneliness and Distress factor. [b] Since the item was included after the translation process, the translation was carried out by the first author using the English-Spanish dictionary www.linguee.es and the translation system www.translate.google.com.mx. [c] The direction of the item was reversed to go in the same direction as the other items of the scale. [d] The item was prepared for IF factor because, in integrating versions Ellonen et al. (2019) and Liel et al. (2019), the factor was left with a single item. It was proposed by the main author. [e] The wording of the item was adapted to be understood and answered by both people with children and people without children. [f] A minor modification of the wording was made to avoid confusion in the respondents. [g] The item was made from one of the response options item 6, being necessary to adapt the wording to use a response scale of 7 points ranging from 1 = strongly disagree to 7 = total agreement. [h] The item was made from one of the response options item 7, being necessary to adapt the wording to use a response scale of 7 points ranging from 1 = strongly disagree to 7 = total agreement. [i] The item was made from one of the response options of item 20, being necessary to adapt the wording to use a scale of responses 8 points ranging from 1 = never to 7 = always, using 0 as Not applicable. [j] The item was made from one of the response options of item 21, being necessary to adapt the wording to use a scale of responses 8 points ranging from 1 = never to 7 = always, using 0 as Not applicable. [k] Due to adaptations made with the other answer options, this translation was not included in later phases.

Appendix B. Family structures of the participants (N = 200)

Family Structure (# of Family members)	f	%	Family Structure (# of Family members)	f	%
Lives alone or without family members (0)	14	7.0	Live only with Father, Mother and 2 children (4)	2	1.0
Live only with Couple (1)	21	10.5	Live only with Father, Mother and 2 relatives (4)	2	1.0
Lives only with one child (1)	9	4.5	Live alone with partner, 2 children and another relative (4)	1	0.5
Lives only with Mother (1)	6	3.0	Live alone with partner and 3 children (4)	3	1.5
Lives only with another relative (1)	2	1.0	Lives only with 3 children and another relative (4)	1	0.5
Live only with Mother and Father (2)	11	5.5	Live only with 4 relatives (4)	2	1.0
Live alone with partner and a child (2)	13	6.5	Lives only with Mother, Father and 3 siblings (5)	7	3.5
Live only with partner and another relative (2)	3	1.5	Lives alone with Mother, Father, 2 siblings and a child (5)	2	1.0
Lives only with 2 children (2)	2	1.0	Lives only with Mother, Father, 2 siblings and a grandparent (5)	2	1.0
Lives only with Mother and another relative (2)	11	5.5	Live alone with Mother, Father, Partner and 2 children (5)	1	0.5
Live only with 2 relatives (2)	2	1.0	Lives alone with Mother, Father and three children (5)	1	0.5
Live only with Mother, Father and a brother / sister (3)	22	11.0	Live alone with partner and 4 children (5)	1	0.5
Lives only with Mother, Father and another relative (3)	2	1.0	Live alone with partner, 2 children and 2 relatives (5)	1	0.5
Lives only with Mother and 2 relatives (3)	11	5.5	Lives only with 5 relatives (5)	2	1.0
Live only with partner and 2 children (3)	9	4.5	Live only with 6 relatives (6)	5	2.5
Lives only with 3 children (3)	1	0.5	Lives only with 7 relatives (7)	3	1.5
Lives only with 3 relatives (3)	2	1.0	Lives only with 8 relatives (8)	1	0.5
Lives only with Father, Mother and 2 siblings (4)	15	7.5	Lives only with 9 relatives (9)	4	2.0
Lives only with Father, Mother, a brother / sister and another relative (4)	3	1.5			
**Total**	**159**	**79.5**	**Total**	**41**	**20.5**

Note. f = Absolute Frequency; % = Relative Frequency

Appendix C. Fit indicators of the confirmatory factorial model of the Spanish translated version of the Balanced Inventory of Desirable Responding with ULSM estimation; indicators of convergent and discriminant validity; discrimination and reliability coefficients (N = 200)

	χ^2	df	$p(\chi^2)$	χ^2/df	CFI	TLI	RMSEA (CI 90%)	p(RMSEA)	SRMR
**Oblique bifactor model**	38.61	26	.053	1.485	.987	.982	.049 (.000, .090)	.049	.049
Orthogonal bifactor model	265.09	27	< .001	9.818	.750	.667	.211 (.185, .237)	< .001	.153
Unifactor model	174.79	27	< .001	6.474	.845	.793	.166 (.135, .199)	< .001	.103

Item	λ (SD)	λ (PH)	r_{tec}
C1. Me resultaría difícil dejar alguno de mis malos hábitos.	.604	---	.451
C3. Cuando tocan mis emociones no puedo pensar adecuadamente.	.641	---	.559
C5. A veces pierdo oportunidades porque no puedo decidirme a tiempo.	.634	---	.512
C9. No siempre sé los motivos de por qué hago lo que hago.	.684	---	.582
C11. A veces digo mentiras si tengo que hacerlo.	---	.727	.626
C12. Hubo ocasiones en las que saqué ventaja de alguien.	---	.764	.682
C13. A veces trato de vengarme en vez de perdonar y olvidar.	---	.687	.561
C14. Dije cosas malas de un amigo a sus espaldas.	---	.632	.580
C15. Recibí más vuelto del que correspondía sin decirle al vendedor.	---	.547	.487

**r (between-factors)**	.505	**AVE**	.534	.581	**Total**
**r^2 (between-factors)**	.255	**α_{Ord}**	.734	.803	.805
		**ω_{Ord}**	.737	.806	.842
		**GLB_{Ord}**	.770	.811	.876

Note. SD = Self-deception; PH = Printing Handling. CFI = Comparative Fit Index; TLI = Tucker–Lewis Index; RMSEA = Root Mean Square Error of Approximation; SRMR = Standardized Root Mean Residual; p = p value; λ = Factor loadings; r_{tec} = Corrected Total-Element Correlation; AVE = Average Variance Extracted; α_{Ord} = Ordinal Cronbach's Alpha Coefficient; CI = Confidence interval; ω_{Ord} = Ordinal McDonald's Omega Coefficient; GLB_{Ord} = Ordinal Greatest Lower Bound Coefficient; r = Pearson's Correlation Coefficient; r^2 = Determination Coefficient.

frontiers

Appendix D. IBCAP-T and PFS-T items in English and Spanish

Inventory Brief Child Abuse Potential Translation (IBCAP-T)		
**Factor**	**English Version**	**Spanish version**
Loneliness	C1. I often feel lonely.	C1. Seguido me siento solo.
	C3. I often feel lonely inside.	C3. Seguido me siento solo por dentro.
	C4. I often feel very lonely.	C4. Seguido me siento muy solo.
	C10. I am often depressed.	C10. Seguido estoy deprimido.
Distress	C6. I often feel very angry.	C6. Seguido me siento muy enojado.
	C7. I get easily angered by my problems.	C7. Me enojo fácilmente por mis problemas.
	C8. I am often angry and I don't know why.	C8. Seguido estoy enojado y no sé por qué.
	C9. I am often angry.	C9. Seguido estoy enojado.
Unhappiness	C11. My life is bad.	C11. Mi vida es mala.
	C12. I am an unhappy person	C12. Soy una persona infeliz
	C13. My life is unhappy	C13. Mi vida es infeliz
Family Conflict	C14. My family has many problems.	C14. Mi familia tiene muchos problemas.
	C15. My family has trouble getting along.	C15. Mi familia tiene problemas para llevarse bien.
	D16. My family fights a lot.	C16. Mi familia pelea mucho.
Impactof Others	C17. People have caused me a lot of pain.	C17. Las personas me han causado mucho dolor.
	C18. Other people have made my life difficult.	C18. Otras personas me han hecho la vida difícil.
	C19. Other people have made my life unhappy.	C19. Otras personas han hecho mi vida infeliz.
Rigidity	C22. Children must be quiet and listen.	C22. Los niños deben de guardar silencio y escuchar.
	C23. Children should never disobey.	C23. Los niños nunca deben desobedecer.
	C24. At home everything must be in its place.	C24. En casa todo debe de estar en su lugar.
	C25. A child needs very strict rules.	C25. Un niño necesita reglas muy estrictas.
Financial Insecurity	C26. Sometimes I worry about not having enough to eat.	C26. A veces me preocupa no tener suficiente para comer.
	C28. I am worried about not having a good income.	C28. Me preocupa no tener un buen ingreso económico.
	C29. Sometimes I spend more money than I should.	C29. A veces gasto más dinero de lo que debería.
	C30. I feel like I have a low income.	C30. Siento que tengo un ingreso económico bajo.

Protective Factors Survey Translation (PFS-T)		
**Factor**	**English Version**	**Spanish version**
Family Functioning and Resilience	D1. The future holds good things for me and my family.	D1. El futuro depara cosas buenas para mí y mi familia.
	D2. The way my family works together helps us deal with difficult times.	D2. La forma en que mi familia trabaja junta nos ayuda a lidiar con los tiempos difíciles.
	D3. In my family we take the time to listen to each other.	D3. En mi familia nos tomamos el tiempo de escucharnos.
	D4. There are activities that are important only to me and my family.	D4. Hay actividades que son importantes solo para mi familia y yo.
Social Supports	I have people I trust to ask for advice on: [D20. Money / Invoices / Budgets]	Tengo personas en las que confío para pedir consejos sobre: [D20. Dinero/Facturas/Presupuestos]
	I have people I trust to ask for advice on: [D21. Relationships and / or my love life]	Tengo personas en las que confío para pedir consejos sobre: [D21. Relaciones y/o mi vida amorosa]
	I have people I trust to ask for advice on: [D22. Food / Nutrition]	Tengo personas en las que confío para pedir consejos sobre: [D22. Alimentación/Nutrición]
	I have people I trust to ask for advice on: [D23. Stress, anxiety, or depression.]	Tengo personas en las que confío para pedir consejos sobre: [D23. Estrés, ansiedad o depresión.]
	I have people I trust to ask for advice on: [D24. Paternity / Children]	Tengo personas en las que confío para pedir consejos sobre: [D24. Paternidad / Hijos]

	I have people I trust to ask for advice on: [D25. Job]	Tengo personas en las que confío para pedir consejos sobre: [D25. Trabajo]
	I have people I trust to ask for advice on: [D26. Housing and / or emergency shelter]	Tengo personas en las que confío para pedir consejos sobre: [D26. Alojamiento y/o refugio de emergencia]
	I have people I trust to ask for advice on: [D27. Education]	Tengo personas en las que confío para pedir consejos sobre: [D27. Educación]
	I have people I trust to ask for advice on: [D28. Legal matters]	Tengo personas en las que confío para pedir consejos sobre: [D28. Asuntos legales]
	I have people I trust to ask for advice on: [D29. Medical / dental care]	Tengo personas en las que confío para pedir consejos sobre: [D29. Atención médica / dental]
Caregivers/ Practitioner Relationship	D17. When I talk to people about my problems, they don't seem to understand me.	D17. Cuando hablo con la gente sobre mis problemas, parece que no me entienden.
	D18. Sometimes I feel like nobody understands me.	D18. A veces siento que nadie me entiende.
	D19. Nobody believes that I can change.	D19. Nadie cree que yo pueda cambiar.
Concrete Supports	In the past six months it was difficult for me to pay: [D38. The bills (electricity, water, gas)]	En los seis meses pasados me fue difícil pagar: [D38. Las cuentas (la luz, el agua, el gas)]
	In the past six months it was difficult for me to pay: [D39. Food]	En los seis meses pasados me fue difícil pagar: [D39. Comida]
	In the past six months it was difficult for me to pay: [D41. Medicines, medical expenses]	En los seis meses pasados me fue difícil pagar: [D41. Medicinas, gastos médicos]
	In the past six months it was difficult for me to pay: [D42. Basic personal or household hygiene items.]	En los seis meses pasados me fue difícil pagar: [D42. Artículos básicos de higiene personal o del hogar.]
	In the past six months it was difficult for me to pay: [D43. Transportation (including gasoline, bus passes, travel)]	En los seis meses pasados me fue difícil pagar: [D43. Transporte (incluido gasolina, pases de autobús, viajes)]
	Last year, due to lack of money, one of the following situations happened to me: [D44. Delay medical or dental care until you have money.]	El año pasado, por falta de dinero, me paso alguna de las siguientes situaciones: [D44. Retrasar la atención médica o dental hasta tener dinero.]
	Last year, due to lack of money, one of the following situations happened to me: [D48. I lost access to my vehicle.]	El año pasado, por falta de dinero, me paso alguna de las siguientes situaciones: [D48. Perdí acceso a mi vehículo.]
	Last year, due to lack of money, one of the following situations happened to me: [D49. I was unemployed when I wanted to work.]	El año pasado, por falta de dinero, me paso alguna de las siguientes situaciones: [D49. Estuve desempleado cuándo quería trabajar.]
